# Bile Acids, FXR, and Metabolic Effects of Bariatric Surgery

**DOI:** 10.1155/2016/4390254

**Published:** 2016-02-24

**Authors:** Olivier F. Noel, Christopher D. Still, George Argyropoulos, Michael Edwards, Glenn S. Gerhard

**Affiliations:** ^1^Temple University School of Medicine, Philadelphia, PA 19140, USA; ^2^Penn State College of Medicine, Hershey, PA 17033, USA; ^3^Geisinger Clinic, Danville, PA 17822, USA

## Abstract

Overweight and obesity represent major risk factors for diabetes and related metabolic diseases. Obesity is associated with a chronic and progressive inflammatory response leading to the development of insulin resistance and type 2 diabetes (T2D) mellitus, although the precise mechanism mediating this inflammatory process remains poorly understood. The most effective intervention for the treatment of obesity, bariatric surgery, leads to glucose normalization and remission of T2D. Recent work in both clinical studies and animal models supports bile acids (BAs) as key mediators of these effects. BAs are involved in lipid and glucose homeostasis primarily via the farnesoid X receptor (FXR) transcription factor. BAs are also involved in regulating genes involved in inflammation, obesity, and lipid metabolism. Here, we review the novel role of BAs in bariatric surgery and the intersection between BAs and immune, obesity, weight loss, and lipid metabolism genes.

## 1. Introduction

Patients with morbid or extreme obesity (BMI > 40 kg/m^2^), who constitute over 6% of the US population, a segment that has increased by 70% from 2000 to 2010 [1], have a prevalence of T2D of over 30% [2]. The prevalence of type 2 diabetes mellitus is increasing in parallel with the increase in obesity. Obesity is associated with a chronic and progressive inflammatory response that is involved in the development of insulin resistance [3–6]. Proinflammatory cytokines and other molecules that are produced by adipocytes, macrophages, and other infiltrating immune cells can disrupt normal insulin signaling and glucose transport inducing insulin resistance [7, 8]. Several antidiabetic drugs may act in part through modulation of adipocyte cytokine production [9], although no drugs have been developed targeting specific adipose derived cytokines and the precise mechanism involved in initiating and mediating the inflammatory processes associated with the pathophysiology of insulin resistance and T2D remains poorly understood. This gap in knowledge is significant since conventional medical management for T2D is only partially effective in achieving acceptable glycemic control and is particularly challenging in obese patients with multiple comorbidities due to metabolic and other related physiological effects.

## 2. Bariatric Surgery

The lack of adequate medical therapies for obesity and T2D has led to the use of bariatric surgery, which is the most effective therapy to induce significant and durable weight loss in patients with extreme obesity (BMI > 40 kg/m^2^) [10–14]. Several types of bariatric surgery, including the commonly performed Roux-en-Y gastric bypass (RYGB) and sleeve gastrectomy (SG) operations, also cause resolution of dysglycemia, insulin resistance, and T2D within hours to days of the procedure, well before weight loss has occurred [15]. This has led to the increasing use of bariatric surgery and to the notion that “bariatric surgery has been shown to be the most effective treatment for obesity and T2DM, both in large well-matched clinical studies and RCTs” [16]. For example, in a single-center, nonblinded, randomized, controlled trial of 60 patients (ages 30–60 years) with a BMI > 35 kg/m^2^ with at least a 5-year history of T2D and a hemoglobin A1c >7%, who were randomly assigned to receive conventional medical therapy or undergo RYGB, no patients in the medical therapy group had achieved remission of T2D, defined as a fasting blood glucose <100 mg/dL and a HGB A1c <6.5% without use of medications at 2 years, whereas 75% of the patients who underwent RYGB were in remission [17]. Perhaps even more striking is the observation that glucose normalization often occurs within hours to days after the surgery, well before any significant weight loss has occurred [11].

Although the metabolic effects of bariatric surgery have been observed for over 50 years [18] and have been confirmed by numerous subsequent studies in humans and animal models [19, 20], the molecular mechanisms underlying these effects are not yet well delineated. For example, initial hypotheses focused on caloric restriction as a mechanism underlying increased glycemic control. Caloric restriction occurs with all bariatric procedures, in which patients undergo essentially identical pre- and perioperative protocols, but not all induce an amelioration of T2D [21]. Indeed, caloric restriction occurs after many surgical procedures [20] but they tend to worsen glycemic control due to the stress response from the surgery [22]. Much attention has also been focused on incretins, particularly glucagon-like peptide-1 (GLP-1). Recent reports using elegant study designs have suggested “a significant role” for GLP-1 in resolution of T2D after RYGB [23, 24], although several reviews [18, 20, 21] attribute only some or none of the antidiabetic effects to GLP-1, consistent with mouse studies in which GLP-1 was not required for either T2D resolution or weight loss after RYGB [25, 26].

The other main classes of hypotheses [20] are based on alterations in the flow and anatomic routing of ingested nutrients [21]. The foregut hypothesis posits that bypass and exclusion from contact with ingested nutrients of the proximal small intestine (foregut), primarily the duodenum, changes the production of a mediator that produces direct anti-T2D effects. Indeed, procedures that cause malabsorption through intestinal bypass have dramatic effects on T2D. This is also supported by the endoluminal sleeve (ES) device, which is endoscopically inserted and prevents ingesta that leaves the stomach from contacting the small intestine for a length similar to that bypassed in RYGB procedures and also results in resolution of T2D [27]. The lower intestinal or hindgut hypothesis is based on the premise that inappropriate delivery of ingested nutrients and/or digestive juices to more distal regions of the small intestine induces a putative molecular mediator that ameliorates T2D. Bile acids have been implicated as key molecules in this hypothesis [18, 21]. Recent work in both clinical studies and animal models supports a key role for bile acids [28–30]. Systemic BA levels are elevated in patients following RYGB [31–34], suggesting an increase in BA signaling after RYGB [35]. However, most studies (reviewed in [21]), including our own [32], have measured levels months after the perioperative period. BAs can regulate cholesterol, triglyceride, and glucose homeostasis [36], making BA regulated pathways attractive molecular targets to various diseases such as T2D, atherosclerosis, obesity, and other diseases [30, 36].

An apparent confounder to this hypothesis is the SG, an operation that is increasingly being used instead of RYGB that is a partial gastrectomy in which the stomach becomes a vertical tube or sleeve. The SG also results in resolution of T2D, which seems to contradict the inappropriate delivery of nutrients and digestive components to the distal intestine hypothesis. However, gastric transit is substantially increased in SG, expediting delivery through the duodenum into the distal intestine [21]. Recently FXR has been shown to be required for the antidiabetic effect of SG in mice [28]. Further delineation of the molecular mechanisms underlying these beneficial effects could provide targets for the development of new nonsurgical treatments [16].

## 3. Novel Roles for Bile Acids in Energy Metabolism

Bile acids are synthesized from cholesterol in the liver [37]. Ingestion of food causes bile acid secretion from the gallbladder through the common bile duct to the duodenum in order to facilitate the absorption of lipids and lipid-soluble vitamins via formation of micelles. Upon reaching the ileum, bile acids are transported by specific transport proteins to the portal circulation for recycling back to the liver. The process is highly efficient with over 95% of bile acids resorbed and the remaining 5% proceeding to the colon and excreting through the stool. Enterohepatic recycling of the bile acid pool occurs about 6–12 times per days; thus the net flux of bile acids through primarily the portal, but also the systemic, circulation is substantial.

**Figure 1:**
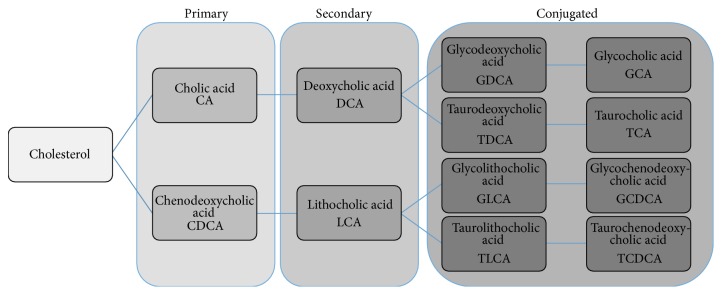
Major bile acids. The primary bile acids are synthesized from cholesterol in the liver. Secondary bile acids are formed by dehydroxylation of primary bile acids by intestinal bacteria. Primary and secondary bile acids can also undergo conjugation with taurine or glycine in the liver.

**Figure 2:**
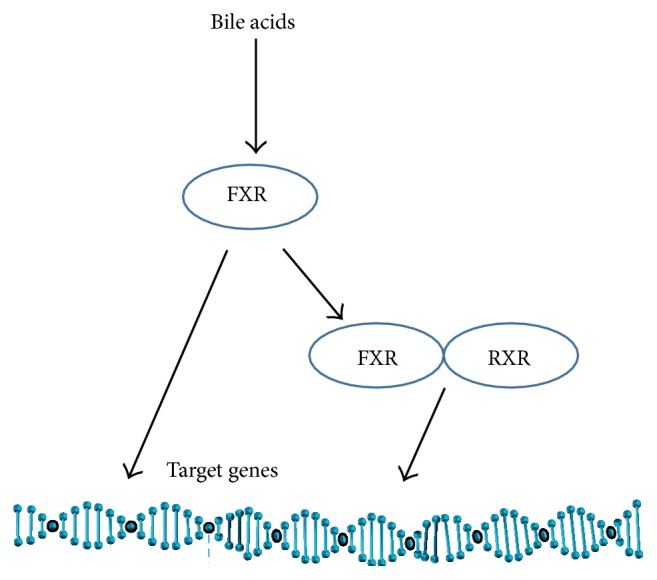
Mechanism of bile acid gene regulation. Bile acids can bind to the farnesoid X receptor (FXR) transcription factor which can regulate gene expression through binding to specific DNA motifs and can heterodimerize with retinoic X receptor-*α* (RXR-*α*) to regulate the expression of target genes.

The two primary bile acids produced by the liver (Figure 1) in humans are cholic acid (CA) and chenodeoxycholic acid (CDCA). These primary bile acids can undergo conjugation with glycine or taurine prior to secretion in the bile to form glycocholic acid (GCA), taurocholic acid (TCA), glycochenodeoxycholic acid (GCDCA), and tauroglycochenodeoxycholic acid (TCDCA). In the intestine, they can undergo dehydroxylation by gut bacteria to produce deoxycholic acid (DCA) and lithocholic acid (LCA). Further chemical modifications can also occur resulting in other minor species. Bile acids can bind to the G-protein coupled TGR5 cellular receptor to mediate signaling [37]. Bile acids also function as a ligand for a specific nuclear transcription factor (Figure 2), the farnesoid X receptor (FXR), which forms a heterodimeric complex with retinoic X receptor-*α* (RXR-*α*) that binds to an inverted repeat sequence in gene promoters [38].

Bile acids not only function in lipid absorption absorbed in the gut but appear to be part of a broader physiological response to ingested nutrients that also involves glucose metabolism [39]. This is consistent with the anabolic need to store fatty acids as triglycerides, which requires a glycerol-3-phosphate backbone that is derived from glucose [40, 41]. Bile acids thus appear to be involved in the regulation of glucose metabolism through modulation of FXR-regulated pathways. FXR^−/−^ mice exhibit peripheral insulin resistance, reduced glucose disposal, and decreased adipose tissue and skeletal muscle insulin signaling, and, conversely, activation of FXR by the agonist GW4064 in insulin-resistant ob/ob mice reduced hyperinsulinemia and improved glucose tolerance [42].

## 4. Metabolism and the Complement Alternative Pathway

One of the inflammatory pathways dysregulated in obesity with a connection to bile acid metabolism is the complement system, a complex network of soluble proteins and membrane receptors largely known for their roles in immunity. The system can be activated to provide immune defense through a stepwise series of proteolytic steps to produce cytolytic complexes. However, there are “sublytic” roles for the complement system in energy metabolism [43]. Activation of complement to fight infections is an energy intensive process; thus dual roles in energy maintenance may have evolved as part of coordinate regulation of immunity and metabolism to manage energy needs [44]. Complement component 3 (C3) is a key complement molecule because it exists in the blood at relatively high concentrations in a nonreactive native form but can be mobilized by three primary activation pathways, the classical, alternate, and lectin, to generate multiple downstream molecules and complexes [45, 46]. Several C3 derived products, including C3a and its corresponding desArg form, are not required for the generation of membrane attack complexes that lyse bacteria.

A potential metabolic role for C3 was first suggested by the finding of low complement levels in lipodystrophy [47]. Adipocytes are known to produce and secrete C3, as well as the alternative pathway activation components Factor B and Factor D, also known as adipsin [48]. Serum levels of C3 thus increase with increasing fat mass and decrease with weight loss [49] and have been implicated in T2D [50]. In the alternative activation pathway, C3 converts spontaneously in plasma to activated C3*∗*H2O which combines with Factor B to form C3*∗*H2OB [51]. Adipsin then cleaves bound Factor B to generate C3*∗*H2OBb. C3*∗*H2OBb is an active convertase that cleaves C3 to generate C3a. C3a can undergo cleavage of the N-terminal arginine by plasma carboxypeptidase B (CpB) to produce C3adesArg, also known as Acylation Stimulating Protein (ASP). ASP binds to C5a like-2 receptor (C5L2 or GPR77) with high affinity [52] and has been shown to stimulate triglyceride synthesis and glucose transport* in vitro* [41, 53, 54]. C5L2^−/−^ mice have higher glucose and insulin plasma levels, with increased expression of insulin resistance genes in adipose tissue [55] and are highly insulin resistant upon glucose tolerance testing [56]. Consistent with the C5L2 knockout mouse findings, administration of ASP to diet-induced obese mice significantly improved glucose tolerance [40].

## 5. FXR and the Complement Alternative Pathway

The regulation of C3 by bile acids via FXR binding motifs has been extensively characterized [57]. We therefore searched the genes that constitute the entire C3-ASP conversion pathway for FXR motifs using MotifMap [58, 59]. The C3, Factor B, Adipsin, CpB, and C5L2 genes all possess predicted FXR binding sites (Table 1). We also observed that the C5L2 promoter contained a V-maf musculoaponeurotic fibrosarcoma oncogene homologue A (MAFA) motif, which plays a key role in the tightly regulated mechanism of induction of insulin gene transcription by glucose [60], suggesting that C5L2 is also regulated by glucose. Further evidence implicating this pathway in glucose metabolism is the observation that adipsin^−/−^ knockout mice exhibit worsened glucose metabolism in diet-induced obesity [61] that recently has been shown to be associated with a role in insulin secretion by pancreatic *β* cells [62].

**Figure 3:**
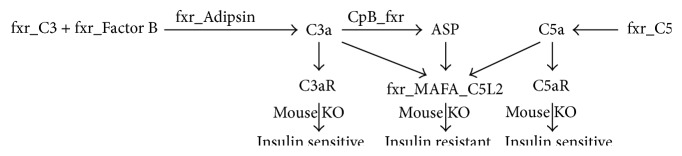
Complement alternative activation pathway with corresponding receptors and mouse knockout phenotypes.

These data support a pathway (Figure 3) in which we hypothesize that the C3 alternative activation pathway is regulated by bile acids to produce ASP that binds to C5L2 to modulate glucose metabolism, consistent with knockout of the C5L2 gene that renders mice insulin resistant.

## 6. FXR and Weight Loss and Obesity Genes

**Figure 4:**
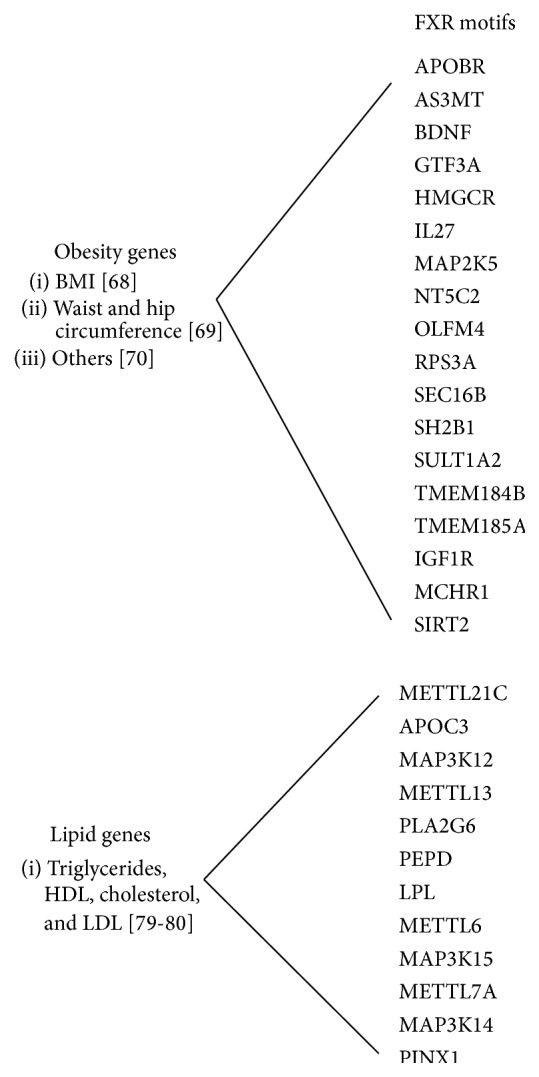
Subset of obesity and lipid associated genes that possess* in silico* identified FXR motifs.

A variety of genetic factors have been identified that associate with weight loss after bariatric surgery including those found through Genome-Wide Association Studies (GWAS) [63–67]. In addition, a recent obesity GWAS of 339,224 individuals identified 97 BMI-associated loci [68] and a GWAS of waist and hip circumference and related traits of 224,459 individuals identified an additional 19 loci new loci [69]. A number of genes have also been associated with Mendelian forms of obesity [70]. We searched all of these genes for FXR motifs using the MotifMap algorithm [58, 71] to identify the following set: APOBR, AS3MT, BDNF, GTF3A, HMGCR, IL27, MAP2K5, NT5C2, OLFM4, RPS3A, SEC16B, SH2B1, SULT1A2, TMEM184B, TMEM185A, IGF1R, MCHR1, and SIRT2 (Figure 4). We then searched PubMed and found evidence of regulation by bile acids for HMGCR [72] and BDNF [73]. We also searched the literature for evidence for whether these genes were expressed in PBMCs and found data for MCHR1 [74], as well as SIRT2 [75] in which expression in PBMCs was modulated caloric restriction [76], and BDNF in which differences in the epigenetic methylation of BDNF were detected in PBMCs in patients who lost weight in a dietary/behavioral weight loss regimen [77]. Decreased levels of BDNF through mutation are associated with obese phenotypes and hyperphagia; thus increasing levels may favor weight loss [78]. Decreasing SIRT2 has been shown to increase *β*-oxidation in adipocytes [75].

## 7. FXR-Regulated Lipid Genes

Triglyceride (TRIG) levels decline and high density lipoprotein cholesterol (HDL-C) levels increase in a majority of patients in the first year following surgery, with a substantial correlation between the degrees of change. We conducted a similar* in silico* search for FXR motifs in a recent compilation of TRIG GWAS genes [79], as well as our own lipid GWAS loci [80], to identify METTL21C, APOC3, MAP3K12, METTL13, PLA2G6, PEPD, LPL, METTL6, MAP3K15, METTL7A, MAP3K14, and PINX1 (Figure 4). Evidence of regulation by bile acids exists for LPL [81]. Interestingly, out of 72 genes, LPL is one of only 10 that were found to be associated with both TRIG and HDL by GWAS. Given the correlation in improvement in TRIG and HDL that we have found following bariatric surgery, LPL is a leading candidate as an FXR-mediated mechanism underlying the improvement in metabolic syndrome related dyslipidemia characterized by high TRIG and low HDL [82].

## 8. Summary

Bariatric surgery has complex effects [20], and thus multiple molecular mechanisms are likely involved. Bile acid signaling through FXR may be common mechanism involved in these effects, though operating through distinct pathways.

## Figures and Tables

**Table 1:** Location of FXR and MAFA consensus binding sequences within 1000 bp of complement alternative activation pathway genes. C3aR and C5aR genes lack either motif.

Gene	Motif	Position	Location
C3	FXR	−154	Upstream
C5	FXR	−454	Upstream
Factor B	FXR	−172	Upstream
Adipsin	FXR	−417	Upstream
CpB	FXR	537	Downstream
C5L2	FXR	−81	Upstream
C5L2	MAFA	−102	Upstream
C3aR	None	—	—
C5aR	None	—	—
